# Patient perceived quality of cirrhosis care– adjunctive nurse-based care versus standard medical care: a pragmatic multicentre randomised controlled study

**DOI:** 10.1186/s12912-024-01934-9

**Published:** 2024-04-19

**Authors:** Maria Hjorth, Anncarin Svanberg, Riccardo LoMartire, Elenor Kaminsky, Fredrik Rorsman

**Affiliations:** 1 Centre for Clinical Research in Dalarna, Uppsala University, Falun, Sweden; 2 Department of Medical Sciences, Uppsala University, Uppsala, Sweden; 3 School of Health and Wellfare, Dalarna University, Falun, Sweden; 4 Department of Public Health and Caring Sciences, Uppsala University, Uppsala, Sweden

**Keywords:** Liver cirrhosis, Nursing care, Quality of care, Pragmatic clinical trial, Multicentre study

## Abstract

**Background:**

Cirrhosis treatment implies prevention and alleviation of serious disease events. Healthcare providers may, however, fail to meet patients’ expectations of collaboration and specific needs of information and support. Individualised nursing care could meet patients’ needs. The aim was thus to measure patient-perceived quality of care after adjunctive registered nurse-based intervention Quality Liver Nursing Care Model (QLiNCaM) compared with standard medical care.

**Methods:**

This pragmatic multicentre study consecutively randomised patients to either adjunctive registered nurse-based care, or standard medical care for 24 months (ClinicalTrials.gov NCT02957253). Patients were allocated to either group at an equal ratio, at six Swedish outpatient clinics during 2016–2022. Using the questionnaire ‘Quality of care from the patient’s perspective’, patients rated their perceived lack of quality for the adjunctive registered nurse-based intervention compared with the control group at 12 and 24 months, respectively.

**Results:**

In total, 167 patients were recruited. Seven out of 22 items in the questionnaire supported the finding that ‘lacking quality’ decreased with adjunctive registered nurse-based care (*p* < 0.05) at 12 months follow-up; however, these differences could not be established at 24 months.

**Conclusion:**

Additional structured registered nurse-based visits in the cirrhosis outpatient team provided support for improved patient-perceived quality of care during the first 12 months. Registered nurses increase patient involvement and present easy access to cirrhosis outpatient care. Patients express appreciation for personalised information. This study reinforces registered nurses’ role in the outpatient cirrhosis team, optimising patient care in compensated and decompensated cirrhosis.

**Trial registration:**

Registered at Clinical Trials 18th of October 2016, [https://www.clinicaltrials.gov], registration number: NCT02957253.

**Supplementary Information:**

The online version contains supplementary material available at 10.1186/s12912-024-01934-9.

## Background

The disease burden of cirrhosis varies during the course of the disease, gradually increasing in the transition from compensated to decompensated phase. Accordingly, the disease will profoundly affect the patients’ everyday life, by decreasing both mental and physical well-being [1], and ultimately also the health-related quality of life [2]. Patients wish for improved collaboration with healthcare providers, personalised cirrhosis information, and care-coordination support [3]. A re-organisation into team-based cirrhosis outpatient care has been suggested, but it is not yet evidence-based [4]. The patients individual expectations, abilities [3], needs [1], and disease knowledge [1, 3] indicate a demand for person-centred cirrhosis care [5, 6]. Despite reports that person-centred nursing care in cirrhosis is likely to improve quality of care for patients with cirrhosis [4, 7, 8], these patients still predominantly receive physician-based outpatient care. One reason for this is the lack of well-defined and evidence-based guidelines regarding cirrhosis outpatient nursing care [9]. Further research regarding the value of structured outpatient clinics led by registered nurses (RN), in addition to standard medical care for patients with cirrhosis, is therefore needed [4, 8, 9].

The World Health Organization [10] broadly defines quality of care as timely and equitable management of healthcare resources. Quality of care is preferably determined by patients’ perception and patient safety. Accordingly, the degree of quality of care has an impact on patients’ health and health economy. Since the highest level of quality may cause disproportionately high costs, improvements in healthcare must be related to its actual costs [11]. Donabedian [11] has operationalised the ‘quality of care’ concept using the terms structure, process, and outcome. The *structure* includes the healthcare organisation, i.e. personnel and materials. The *process* comprises activities in the patient-care provider interaction. The *outcome* is for instance, impacts on patients’ satisfaction with care, which is highly relevant, since patients may value quality of care differently compared with the healthcare organisation. Patient experiences may be explored by patient-reported experience measures (PREM). Patient perspectives on quality of care have been explored by Wilde et al. [12], which is further understood in two conditions, namely the resources in the care organisation, and the degree to which patient’s wishes are met. Traditionally, in cirrhosis care, clinical outcomes of healthcare have involved survival rates, disease progression, symptom management [7, 13–15], or medical quality indicators on an organisational level [14]. Previous validations of cirrhosis care have not included patient-reported outcomes on satisfaction of health care services [8, 9], i.e. PREM [11, 13], which is closely related to patient safety [13]. The questionnaire ‘Quality of care from the patient’s perspective’ (QPP), developed by Wilde et al. [12, 16], enables evaluation of PREM in a broad variety of outpatient settings. Further, QPP may distinguish ‘lacking quality’ from balanced or excess quality service, per item or within its four domains: medical-technical competence, identity-oriented approach, socio-cultural atmosphere, and physical-technical conditions [17].

To meet patients’ varied needs in cirrhosis illness [1], a multi-disciplinary care approach that involves nursing care is recommended [4, 7, 8]. It remains unclear if cirrhosis nursing care is as beneficial in addressing individual patient needs as reported in other chronic medical conditions [18, 19]. Previous attempts of evaluating self-care models for cirrhosis outpatient care shed light on the complexity in finding the core of cirrhosis self-care programmes and appropriate outcome measurements [8, 9]. Therefore, pragmatic and well-defined interventions with patient-oriented outcomes are prompted to improve cirrhosis care [8]. Despite PREMs providing important insights from the patient perspective in the development of new care models [13], patients’ opinions are rarely considered as outcomes in outpatient RN-based interventions in cirrhosis. Therefore, to address shortcomings of the standard medical cirrhosis outpatient care [3], the implementation of structured RN-based programmes for persons living with cirrhosis [20] is highly relevant and should be followed by patient-related outcomes, such as PREMs [11]. Accordingly, this study aimed to compare patient-perceived quality of cirrhosis care after receiving either adjunctive RN-based intervention, the Quality Liver Nursing Care Model (QLiNCaM), or standard medical care.

## Methods

### Design

This pragmatic, multicentre, randomised parallel group trial evaluated the effectiveness of an adjunctive RN-based intervention on patient-perceived quality of cirrhosis outpatient care. Patient-perceived quality of care was a secondary outcome measure of the adjunctive RN-based intervention. The primary outcome health-related quality of life will be reported in a separate future publication. The entire study population contributed with data for all outcome measures. We hypothesized that the perceived ‘lacking quality’ of cirrhosis care would decrease with QLiNCaM compared to standard medical care. The study adhered to the Consolidated Standards of Reporting Studies (CONSORT) statement [21] (Additional file 1), and was conducted as part of a larger project that targeted different domains of adjunctive RN-based care, QLiNCaM. Full details of the study design and methodology have been reported previously (ClinicalTrials.gov NCT02957253), with available statistical analysis plan. The intervention and its outcomes have been described in detail in a study protocol [20].

### Settings and patients

In Sweden, cirrhosis care is mainly medical and physician-based at outpatient settings, present at both county and university hospitals. This study was conducted at six outpatient clinics in mid- and south Sweden, two county hospitals, and four university hospitals, from November 2016 to December 2022. One physician and one to four RNs per study site were responsible for recruitment and allocation of participating patients, as well as data collection. At each study site, one or two RNs (hereinafter referred to as intervention nurses; INs), were responsible for the delivery of the intervention. The IN’s role and training are described in detail below.

The sample size was calculated based on the primary outcome of the entire project; thus, QPP was not preceded by a power calculation [20]. Eligible patients were aged 18–85, diagnosed with cirrhosis within the last 24 months, and planned for standard medical outpatient care. Diagnosis of cirrhosis was based on clinical grounds, i.e. clinical signs, laboratory findings, histology, magnetic resonance imaging, computer tomography, ultrasound and/or transient elastography. Non-Swedish speaking patients, those with persistent hepatic encephalopathy or severe comorbidities were excluded [20]. Patients’ eligibility assessment took place at ordinary outpatient visits with the physician or at discharge from inpatient care. Following informed consent and completion of baseline measurements, patients were consecutively allocated by use of a concealed computerised randomisation sequence [Randomize.Net, Interrand, Ottawa, Canada]. The patients were randomised at a 1:1 allocation ratio to either receiving the QLiNCaM intervention or standard medical care. The allocation sequence used random block sizes of four, six, and eight and was stratified for study centre and disease severity (compensated vs. decompensated cirrhosis). The individual study participation was 24 months (± 2 months). Dedicated INs and RNs at each study site were responsible for data collection at enrolment and at 12 and 24 months of follow-up for the intervention and control group, respectively [20].

### Intervention

**Fig. 1:**
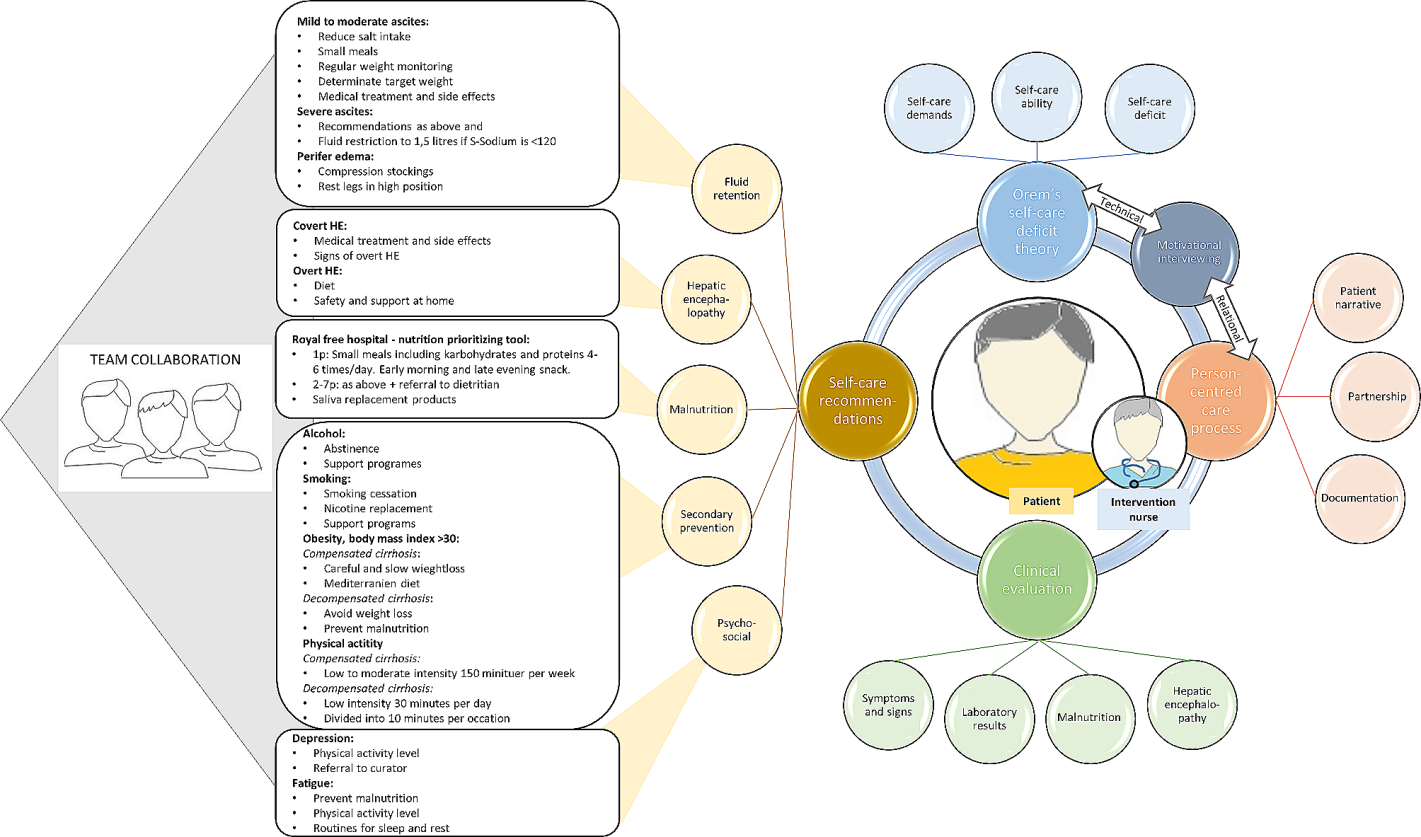
Description of the QLiNCaM intervention in terms of decision support, self-care recommendations, and team collaboration. For clinical evaluation of malnutrition the Royal Free Hospital - Nutritional Prioritising Tool [24]was used. Hepatic encephalopathy was assessed by use of psychometric tests [25, 26] and the West Haven criteria [34]

The fundaments of the QLiNCaM intervention previously described as ‘adjunctive nursing care based on Orem’s nursing theory’ [20] were person-centred nursing care according to Ekman et al. [22] in addition to Orem’s self-care deficit theory of nursing [23], in order to strengthen patients’ independent self-care abilities. This was, for example, about improving knowledge of self-care with regard to the degree of health literacy, promoting physical and cognitive abilities to engage in self-care, increasing motivation for self-care and involving the patient in decision-making. The person-centred self-care recommendations were based on the patients’ narrative. Furthermore, the INs evaluated signs and symptoms of disease deterioration, laboratory findings, and screening instruments for early detection of malnutrition [24] and covert hepatic encephalopathy [25, 26] (Fig. 1). Motivational interviewing [27] was used as a mediator of person-centred communication. The intervention was initiated in 2016 on the prevailing evidence and clinical praxis for cirrhosis outpatient care regarding: (I) fluid retention [28]; (II) hepatic encephalopathy [29]; (III) malnutrition [24, 30]; (IV) secondary prevention [31, 32]; and (V) psycho-social aspects of cirrhosis illness [33]. If needed, INs had the possibility to refer patients to other healthcare professions in the team, such as a physician, dietitian, physio-therapist or social counselor (Fig. 1). The INs received training in the QLiNCaM intervention and motivational interviewing before participant recruitment. To increase intervention concordance throughout the course of the study, four tutorial group sessions were accomplished during 2017 to 2019, gathering the INs from all study sites. The study protocol [20] scheduled patients in the compensated cirrhosis phase to visit INs once a year, whereas patients in the decompensated phase were offered visits up to twice a month. The intervention was added to the Swedish standard medical care, as previously described in the study protocol [20].

### Control group

The control group had no or limited contact with the INs, and members of the group were primarily cared for by physicians and RNs according to the standard of care. The standard medical care included telephone calls to RNs on demand, outpatient visits and telephone contact with physicians, screening programme for hepatocellular carcinoma, endoscopy for variceal control, and laparocentesis for ascites. The frequency of visits in the control group was not influenced by the study.

### Outcome measures and data collection

Quality of care was defined according to Wilde et al. [12]: ‘the resource structure of the care organisation’, i.e. perceived reality, and ‘the patient’s preferences’, i.e. subjective importance. Each item contained two aspects: (I) *perceived reality* of care worded as ‘This was what I experienced…’ and (II) *subjective importance* of the item worded as ‘This is how important it was to me…’. Each condition was rated on a four-point Likert scale ranging from one (do not agree/of little or of no importance) to four (fully agree/of very high importance). In the absence of experience in an item, each item had a fifth response option, ‘not applicable’ [12, 16]. According to the QPP manual [17], *perceived reality* and *subjective importance* were used to convert item responses into a seven-point score. ‘Lacking quality’ was defined as the two lowest scores per item, i.e. substandard or poor quality, which was the study outcome. QPP has been psychometrically evaluated [12] and validated in its four domains, per item [16, 35], and also for electronic use [36].

An electronic short form [16, 36] of the original QPP questionnaire [12], containing 22 items, was used (Additional file 2), of which 17 were original QPP items (item 5 to 21) and five modified items (item 22 to 26). The modified items were added by the research group in agreement with the instrument developer, as described in the study protocol [20]. Furthermore, these items are not part of the original QPP but follow the same structure as QPP in wording and response options. The 17 original QPP items covered three of the original four QPP dimensions: *medical-technical competence* (3 items), *identity-oriented approach* (10 items), and *socio-cultural atmosphere* (4 items). Items belonging to the fourth domain, *physical-technical conditions*, were removed due to low relevance in outpatient cirrhosis care.

Data collection also concerned decompensation episodes, number of outpatient visits and admissions to hospital, laboratory sampling for Child Pugh score, and MELD score [37]. Hepatic encephalopathy was determined with the psychometric hepatic encephalopathy score [25], continuous reaction time [26], and West-Haven criteria [34]. Risk of malnutrition was assessed using Royal Free Hospital-Nutritional Prioritising Tool [24] and health literacy with the Newest Vital Sign [38].

### Data analysis

In line with the a priori statistical analysis plan (ClinicalTrials.gov NCT02957253) and the study protocol [20], the complete cases were analysed according to intention-to-treat principles, where patients are coded to their allocation status irrespective of intervention received. The patients stated lack of quality of care was compared per QPP item between the intervention and control groups, using Firth’s penalised logistic regression (logistf v1.24.1 in R v4.2.3), for 12- and 24-months follow-up data [39]. The model was adjusted for baseline disease severity (compensated or decompensated phase). Estimates were presented as odds-ratios combined with 95% confidence intervals, and hypothesis tests were based on profile penalised likelihood [39].

## Results

### Patients’ characteristics

**Fig. 2:**
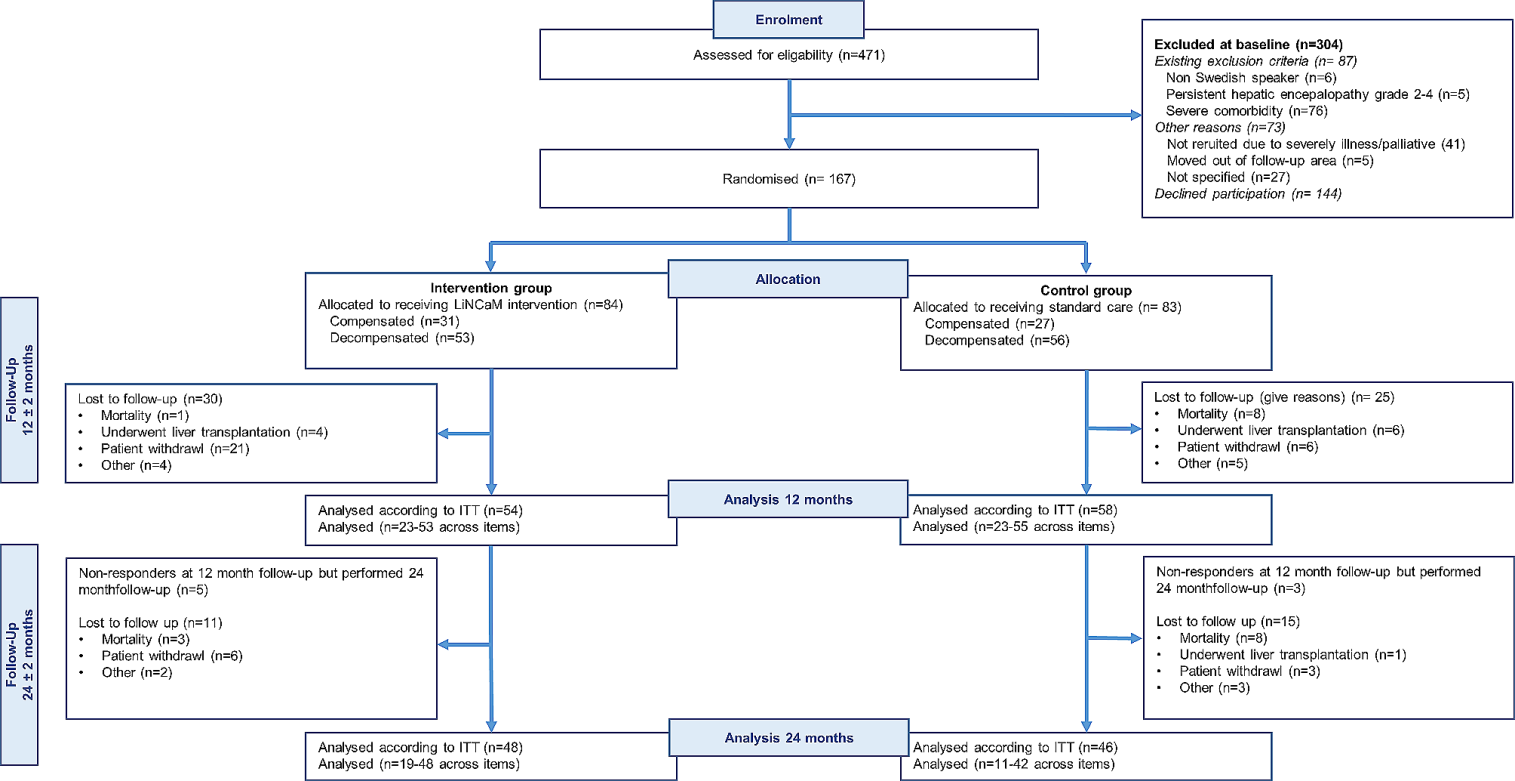
Enrolment process and allocation flow chart

Of the entire cohort of 167 enrolled patients in this randomised controlled study, 84 were allocated to the intervention group and 83 to the control group (Fig. 2). At baseline, 109 of the 167 patients (65%) had experienced at least one episode of cirrhosis decompensation (Fig. 2). Diabetes was the most frequent co-morbidity (intervention *n* = 16; control *n* = 11), followed by cardiovascular disease (intervention *n* = 7; control *n* = 9). One hundred-and-twelve of the 167 patients (67%) completed the QPP questionnaire at 12 months follow-up and 94 (56%), at 24 months follow-up (Fig. 2; Table 1).

**Table 1:** Characteristics of participating patients. At time of enrolment, 12 months and 24 months; and consumed outpatient and inpatient care during study participation

Characteristics	Enrolment		12 months		24 months	
	Intervention group (*N* = 84) n (%)	Control group (N = 83) n (%)	Intervention group (N = 54) n (%)	Control group (N = 58) n (%)	Intervention group (N = 48) n (%)	Control group (N = 46) n (%)
Female gender	37 (44)	35 (42)	24 (44)	26 (45)	20 (42)	21 (46)
Age (years)						
18–39	2 (2)	3 (4)	0	2 (3)	0 (0)	1 (2)
40–65	40 (48)	38 (46)	23 (43)	24 (42)	22 (46)	19 (41)
65–85	42 (50)	42 (50)	31 (57)	32 (55)	26 (54)	26 (57)
Country of birth						
Sweden	78 (93)	76 (91)	51 (94)	54 (94)	45 (94)	43 (94)
Europe	5 (6)	3 (4)	3 (6)	2 (3)	3 (6)	1 (2)
Other	1 (1)	4 (5)	0	2 (3)	0 (0)	2 (4)
Education						
Elementary school	23 (27)	19 (23)	18 (33)	14 (24)	15 (31)	12 (26)
Upper secondary school	41 (49)	41 (49)	24 (45)	25 (43)	23 (48)	22 (48)
University	20 (24)	23 (28)	12 (22)	19 (33)	10 (21)	12 (26)
Occupation						
Working/studying	17 (21)	15 (18)	8 (15)	10 (17)	10 (22)	7 (15)
Retired	39 (47)	43 (52)	28 (52)	33 (57)	23 (48)	28 (62)
Disability pension	4 (5)	2 (3)	4 (7)	2 (3)	3 (6)	1 (2)
Non-employed	8 (9)	3 (4)	3 (6)	1 (2)	1 (2)	1 (2)
Partially sick-leave	8 (9)	8 (9)	4 (7)	4 (7)	5 (10)	1 (2)
Full time sick leave	1 (1)	4 (5)	1 (2)	3 (5)	1 (2)	3 (6)
Other occupation	7 (8)	8 (9)	6 (11)	5 (9)	5 (10)	5 (11)
Cirrhosis diagnosis						
Alcohol related liver disease	48 (57)	38 (46)	27 (50)	28 (48)	23 (47)	18 (39)
Autoimmune hepatitis	6 (7)	7 (8)	6 (10)	6 (10)	6 (13)	6 (13)
Viral hepatitis C	4 (5)	3 (4)	2 (4)	1 (2)	2 (4)	1 (2)
Non-alcoholic fatty liver disease	14 (17)	8 (9)	10 (19)	6 (10)	10 (20)	5 (11)
Cryptogenic	9 (11)	20 (25)	8 (15)	12 (21)	6 (12)	12 (26)
Other^†^	3 (3)	7 (8)	1 (2)	5 (9)	1 (4)	4 (9)
Child Pugh group						
A	52 (62)	47 (57)	39 (72)	36 (62)	31 (65)	30 (65)
B	26 (31)	31 (37)	14 (26)	22 (38)	15 (31)	16 (35)
C	6 (7)	5 (6)	1 (2)	0 (0)	2 (4)	0 (0)
MELD score						
< 10	44 (52)	41 (49)	31 (57)	31 (54)	24 (50)	22 (48)
10–15	29 (35)	33 (40)	20 (37)	25 (43)	21 (44)	22 (48)
> 15	11 (13)	9 (11)	3 (6)	2 (3)	3 (6)	2 (4)
Drugs^‡^						
Diuretics	44 (52)	44 (53)	27 (50)	29 (50)	24 (52)	21 (46)
Lactulose	33 (39)	35 (42)	17 (31)	23 (40)	17 (37)	17 (37)
Rifaximin	10 (12)	4 (5)	5 (9)	2 (3)	5 (11)	2 (4)
non-selective beta-blockers	32 (38)	24 (29)	22 (41)	15 (26)	20 (44)	8 (17)
Comorbidity						
None	50 (60)	49 (59)	29 (54)	33 (57)	27 (56)	27 (59)
1–2	34 (40)	30 (36)	25 (46)	21 (36)	21 (44)	15 (33)
> 2	0 (0)	4 (5)	0 (0)	4 (7)	0 (0)	4 (8)
Outpatient care^§^						
Physician	N/A	N/A	69 (1.2)	81 (1.4)	62 (1.3)	53 (1.2)
Intervention Nurse	N/A	N/A	140 (2.6)	N/A	135 (2.8)	N/A
Inpatient care^§^						
Numbers of admissions	N/A	N/A	28 (0.5)	17 (0.3)	30 (0.6)	25 (1)
Days at hospital	N/A	N/A	78 (1.3)	178 (3)	87 (1.7)	116 (2)
Other team members						
Dietitian	N/A	N/A	14 (26)	7 (12)	5 (10)	8 (17)
Physiotherapist	N/A	N/A	5 (9)	1 (2)	3 (6)	1 (2)
Social counsellor	N/A	N/A	4 (7)	3 (5)	6 (13)	2 (4)

^†^ LC due to toxicity, cardiaque, primary biliary cholangitis or α-1 antitrypsinemia

^‡^ Missing data at 12 months (*n* = 4), 24 months (*n* = 3

^§^ Reported in total care needs for each group with mean of number of events in brackets

**Fig. 3:**
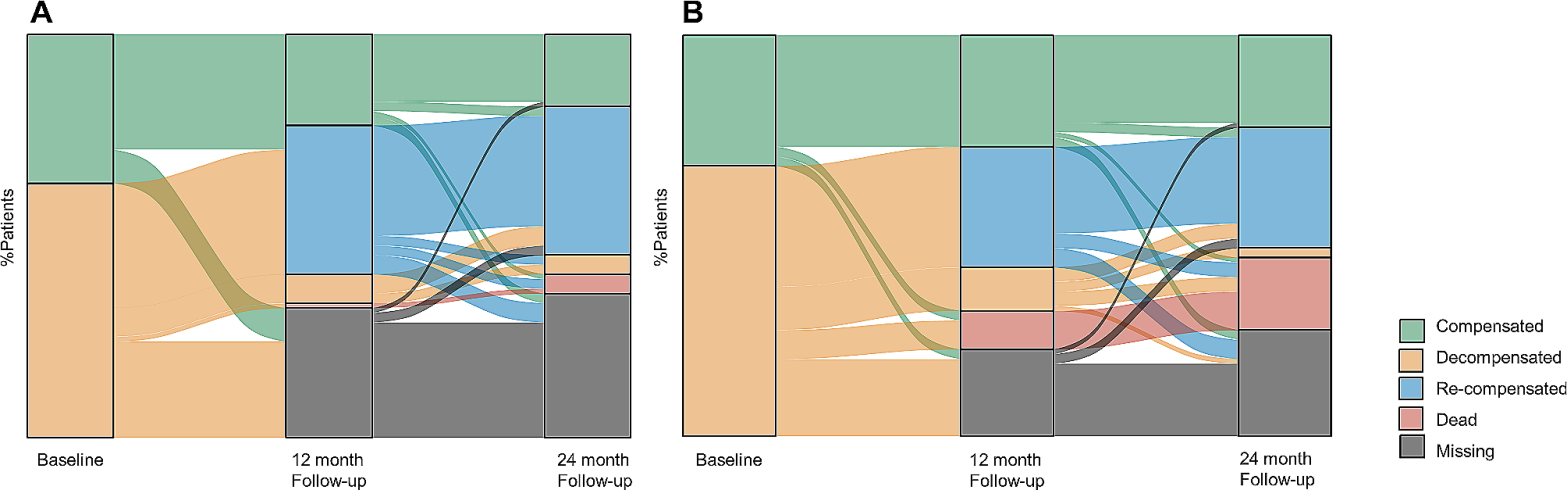
Alluvial plot of disease state transitions per time point and proportion of missing data. (**A**) Intervention group and (**B**) Control group

Patients’ characteristics at baseline, 12 months follow-up, and 24 months follow-up are presented in Table 1. The severity of the patients’ cirrhosis disease varied during the course of the study. Fifteen patients had unchanged and stable disease severity, i.e. compensated, throughout the study, whereas the disease deteriorated from compensated to decompensated in 43 patients. After decompensation, the disease stabilised, improved, or re-compensated in some of the patients. The mortality rate was four and 16 in the intervention group and control group, respectively (Fig. 3A and B).

### Effectiveness of the QLiNCaM intervention on patient’s perceived quality of cirrhosis care

**Fig. 4:**
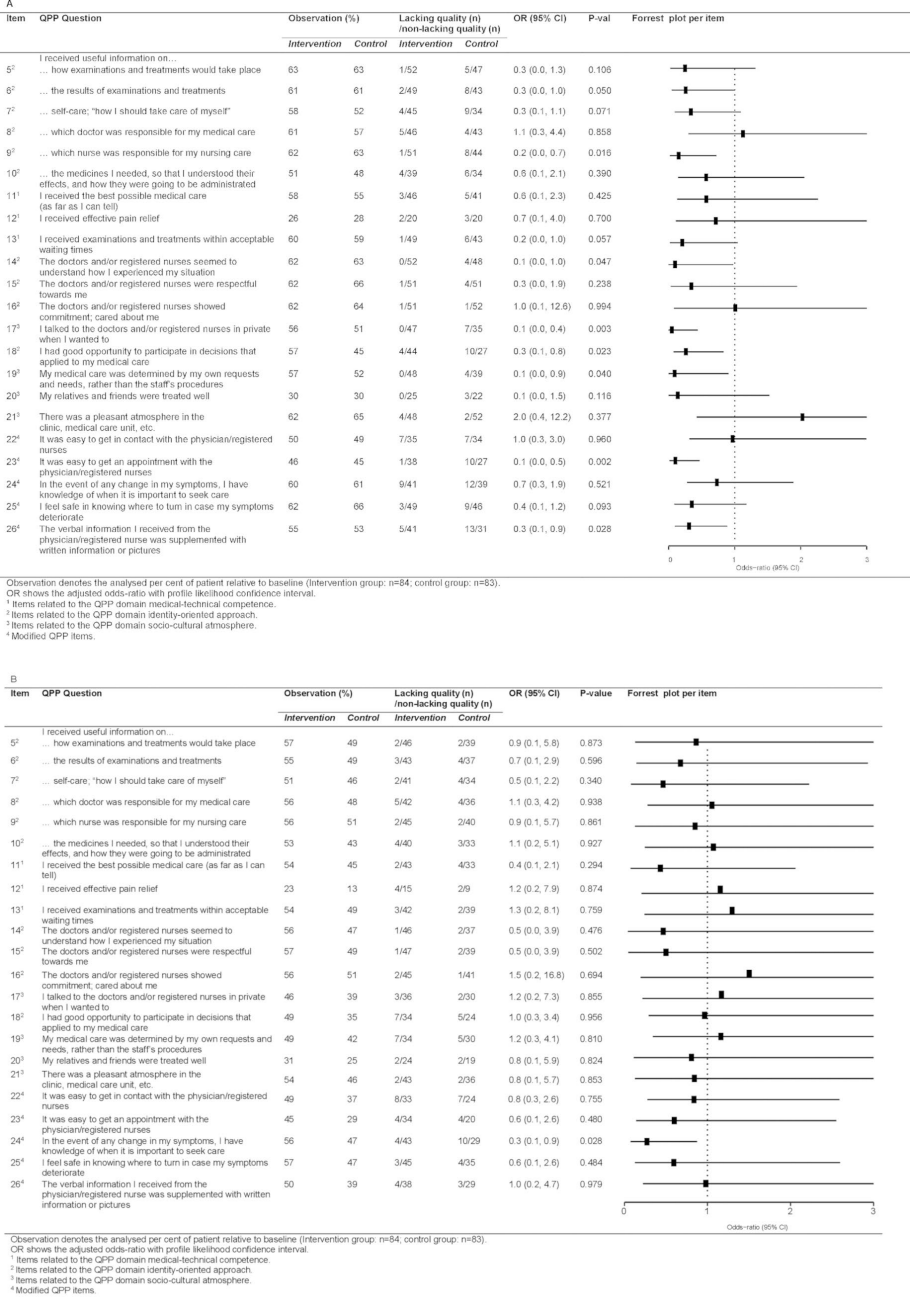
Comparison of patient-perceived ‘lacking quality’ between intervention study groups per QPP item. (**A**) 12 months and (**B**) 24 months follow-up. Odds-ratio of ‘lacking quality’. Squares denote point estimates and error bars their 95% confidence interval. The dotted vertical line indicates odds-ratio = 1. Odds ratio below one proves a positive effect; odds ratio larger than one demonstrates a negative effect of the intervention

After 12 months, seven out of 22 items in the questionnaire supported the finding that ‘lacking quality’ was reduced with QLiNCaM (Fig. 4A). Three of the items belonged to the QPP dimension *identity oriented approach*: (I) ‘having a responsible RN’; item nine (OR 0.2, 95% CI 0.0–0.7), (II) ‘doctors/RNs seemed to understand how I experienced my situation’; item 14 (OR 0.1, 95% CI 0.0–1.0), and (III) ‘having opportunity to participate in decisions that applied to medical care’; item 18 (OR 0.3, 95% CI 0.1–0.8). Two items belonged to the QPP domain *socio-cultural atmosphere*: (I) ‘conversations were held in privacy’; item 17 (OR 0.1, 95% CI 0.0–0.4), and (II) ‘receiving health care determined by own requests and needs rather than the staff’s procedures’; item 19 (OR 0.1, 95% CI 0.0–0.9). Two of the modified items also demonstrated a significant difference in ‘lacking quality’ between the intervention group and the control group: I) ‘access to receive visiting time’; item 23 (OR 0.1, 95% CI 0.0–0.5) and ‘receiving written information in supplement to verbal information’; item 26 (OR 0.3, 95% CI 0.1–0.9). At 24 months, there was no difference between the intervention group and the control group (Fig. 4B). Additional file 3 provides a complete and detailed description of the analysis.

### Received care during participation

During the 24 months of participation, the total number of outpatient visits (physician visits and RN visits combined) tripled (mean = 4.0) in the intervention group compared with the control group (mean = 1.3). Conversely, the days within inpatient care almost doubled in the control group compared with the intervention group (mean 2.5 vs. 1.5) (Table 1).

Twelve of the participants in the intervention goup droped out before receiving the intervention. The reasons were voluntary or due to severe illness and liver transplantation (Fig. 2). The remaining 72 participants in the intervention group made 275 visits to INs during the 24-month follow-up (mean 2.7 per patient and year of study participation). Seventy-three per cent of the visits were independently performed by the INs. The major reasons for physician consultations were medical questions, e.g. treatment of cirrhosis complications (*n* = 30), drug prescription (*n* = 18) or treatment of comorbidities, such as cardiovascular disease or pain conditions (*n* = 17). Patients in the control group received sporadic RN visits, 30 visits, in total, during the first 12 months (mean = 0.5), and 10 visits during the second 12 months (mean = 0.2).

Due to the Covid-19 pandemic during 2020–2022, data collection was periodically restricted to telephone contacts and questionnaires sent by mail (with prepaid reply envelopes) for 24 of the patients at the 12 months follow-up (intervention *n* = 11; control *n* = 13), and 30 of the patients at the 24 month follow-up (intervention *n* = 8; control *n* = 22). During the study period (2016–2022), there was a growing interest and awareness of RN involvement in the outpatient cirrhosis care in Sweden, which made it impossible to further extend the study for the inclusion of additional patients.

## Discussion

This study compared patient-perceived quality of cirrhosis care after receiving either adjunctive RN-based care (QLiNCaM) or standard medical care in a Swedish outpatient setting. The results of our study disclose a need for structured RN-based clinics in outpatient cirrhosis care. Importantly, patients in the intervention group stated there was improved accessibility to outpatient cirrhosis care. They also thought the information received was useful and adapted to their needs. Furthermore, the patients responded that they had been involved in decisions regarding their care to a higher extent in the intervention group, aspects that were in line with the fundaments of the intervention (Fig. 1). This strengthens the independent role for RNs in the cirrhosis team to provide support and meet patients’ needs of information [1, 3] and wishes for improved cirrhosis outpatient care [3]. To our knowledge, this is the first study presenting patient-reported experience measures from a RN-based intervention in cirrhosis care. Previous studies have mainly addressed organisational and process components regarding quality aspects of cirrhosis care [7–9, 14, 15].

The statistically significant changes in patient-reported ‘lacking quality’ in seven out of 22 QPP items at 12 months follow-up, are striking, and contradict previous criticism raised against RN involvement in cirrhosis management [9]. The significant improvements in QPP items 14, 18, and 19 (Fig. 4A) concern patients’ reports of being understood, invited to participate and have their needs and requests taken into account in meetings with healthcare providers, thus facilitating a person-centred partnership and caring with empathy by patients’ needs [5, 6]. The patients’ experience of cirrhosis illness as an unpredictable condition [1] and the variation in patients’ needs due to disease severity (Fig. 3A, B) reinforce the need for a person-centred cirrhosis care. Altogether, the intervention facilitated patients to feel secure in the continuum of cirrhosis care, which has, in line with results of nursing care in cancer [18], been recognised as elemental in the care of patients with cirrhosis [3]. After two years, the effectiveness of the intervention could not be proven better than standard of care (Fig. 4B), in terms of “lacking quality” of care.

This study demonstrates that the intervals between RN visits have to be based on the patient’s actual needs. Accordingly, some patients occasionally need frequent visits, whereas visits once yearly are relevant in others. Since recommendations on frequency of visits to RNs in cirrhosis outpatient care are lacking [4, 8], our findings may provide guidance on visit intervals in a mixed patient population with compensated and decompensated cirrhosis. However, the increased use of outpatient care, and the differences in the need of inpatient care between the two study groups, motivates a future health economic evaluation of the intervention [20].

In line with the Code of Ethics for Nurses [46], this study highlights the important role that independent RNs have in the healthcare team to improve quality of care and safety for patients with cirrhosis (Fig. 1).

### Strengths and limitations

This pragmatic prospective study was performed in clinical practice with six participating study sites. The large number of patients with omitted results may imply a risk for selection bias. However, the similar distribution of measurement for patient characteristics across the three data collection time points (Table 1) provides support for internal validity of the results. Therefore, comparison between the intervention and control groups was considered reasonable.

As far as we know, this is one of the largest populations with longitudinal data after receiving RN-based intervention in cirrhosis [8]. Regarding aetiology, age, gender, and underlying liver disease, the study population is representative compared with previously studied cirrhosis populations in Sweden [45]. Another strength is the pursuit of a consistent study population, only patients with a cirrhosis diagnosis ≤ 24 months included in the study. Although this limited the number of eligible patients, the intention was to reduce selection bias [41]. Another robustness of this study is the detailed description of the QLiNCaM intervention [20], which enables replication among other cohorts [41]. Further, the effectiveness of the intervention applied to both compensated and decompensated cirrhosis, in line with recently published recommendation regarding RN-based cirrhosis outpatient care [4]. The implementation strategies, e.g. IN group tutorials, assured proper and equal accomplishment of the intervention at all six study sites. Therefore, as previously proposed [4, 8, 9], we consider the QLiNCaM intervention as a model for future clinical nursing interventions in cirrhosis.

In the planned recruitment process, we calculated for 33% non-included patients. The actual number was almost dubbled, i.e. 64% (Fig. 2). More patients than anticipated declined participation or chose to withdraw from the study, which reduces the possibility for generalisation of the study results. However, the pragmatic design increases generalisability into clinical settings [44]. Missing data due to mortality during the 24 month follow-up period is less than what is experienced in other prospective intervention studies in outpatient cirrhosis populations [15, 40]. However, the populations in those studies were patients with previous episodes of decompensation, or were limited to patients with alcohol-related cirrhosis [40]. In the present study, many patients in the intervention group unfortunately chose to discontinue their participation, and it can be speculated that the intervention may have been a burden and the reason for withdrawal per se. Contrary, in the control group, mortality and liver transplantation were common reasons for withdrawal. This may have contributed to a selection bias, reducing the internal validity [41].

Intervention contamination may have attenuated our effects, since each clinic simultaneously provided both QLiNCaM and standard medical care. When designing this study, the 24-month study length was carefully considered as appropriate to identify effects of the intervention. However, in patients with cirrhosis, and particularly in decompensated disease, the prognosis is poor, with a median survival time of approximately two years [19]. A 24-month follow-up may therefore not be appropriate and result in a lot of missing data. The Covid-19 pandemic not only hampered the inclusion of patients in the study, but the planned procedure for data collection also had to be changed to telephone contacts instead of outpatient clinic visits for some of the patients that affected the data collection. Further, several patients were sent paper QPP questionnaire by mail that also restricted data collection and completeness of questionnaire responses. To enhance participant recruitment and complete the data collection in included participants, repeated dialogues were held with the first line managers, RNs and INs.

The original QPP questionnaire [12, 16] contains four domains, of which the used electronic QPP questionnaire excluded the physical-technical domain. Nevertheless, relevant questions were prioritised, which outweighed the risk of low response rates due to non-relevant questions. In turn, this could be a threat to the reliability of the electronic QPP questionnaire used in this study. However, our analysis was based on items, not domains. Firth’s logistic regression [39] was used in place of the originally planned logistic regression (ClinicalTrials.gov NCT02957253) [20] due to fewer outcome events than anticipated and to avoid convergence problems. It is a recommended method to manage bias in small samples by shrinking effects towards zero [39, 42]. Nonetheless, low power as a result of small sample size is known to increase uncertainty in estimates and consequently also the risk of false negatives (i.e. type-II error). Despite the considerably small sample, seven out of 22 QPP items were statistically significant at 12 months follow-up, which is more than could be expected by chance [41]. However, the intervention effect on QPP still has to be interpreted with caution due to the risk of attrition-related selection bias.

### Implications

RNs have a role in cirrhosis outpatient care to improve patient-perceived quality of care and to realise a person-centred care approach. RNs should therefore be considered as a resource in caring for the growing cirrhosis population, and be part of the interdisciplinary cirrhosis care team. The present results pertain to a Swedish cirrhosis outpatient healthcare context. However, the detailed description of the intervention in this study enables replication of the study in other healthcare organisations. The QLiNCaM intervention may also be applicable to RN interventions in other chronic illnesses after adjustments on disease specific evaluations and self-care recommendations. We encourage future studies to validate our results.

## Conclusions

In comparison to standard medical care, this study indicates that adjunctive RN-based care improves patient-perceived quality of care by increasing patients’ involvement in their healthcare, and by improving access to cirrhosis outpatient care. Patients express appreciation for personalised information. Altogether, we believe that structured RN involvement have a potential to play an important role and make a difference for patients’ sense of safety in the continuum of cirrhosis care.

## Electronic supplementary material

Below is the link to the electronic supplementary material.

Supplementary Material 1

Supplementary Material 2

Supplementary Material 3

## Data Availability

Data are available from Region Dalarna upon reasonable request (e-mail: forsknings.utlamnande@regiondalarna.se) provided that tha data can be made available in accordance with applicable data protection and privacy regulations.

## Abbreviations

IN: Intervention nurse
RN: Registered nurse
PREM: Patient-reported experience measures
QLiNCaM: Quality Liver Nursing Care Model
QPP: Quality of care from the patient’s perspective
