# Correction: Patient perceived quality of cirrhosis care– adjunctive nurse-based care versus standard medical care: a pragmatic multicentre randomised controlled study

**DOI:** 10.1186/s12912-026-04350-3

**Published:** 2026-01-29

**Authors:** Maria Hjorth, Anncarin Svanberg, Riccardo LoMartire, Elenor Kaminsky, Fredrik Rorsman

**Affiliations:** 1 Centre for Clinical Research in Dalarna, Uppsala University, Falun, Sweden; 2 Department of Medical Sciences, Uppsala University, Uppsala, Sweden; 3 School of Health and Wellfare, Dalarna University, Falun, Sweden; 4 Department of Public Health and Caring Sciences, Uppsala University, Uppsala, Sweden

Correction: BMC Nurs. **23**, 251 (2024). https://doi.org/10.1186/s12912-024-01934-9

Following the publication of the original article [1], it was noted that Fig. 4b was missing and should have appeared as shown below.

**Fig. 4:**
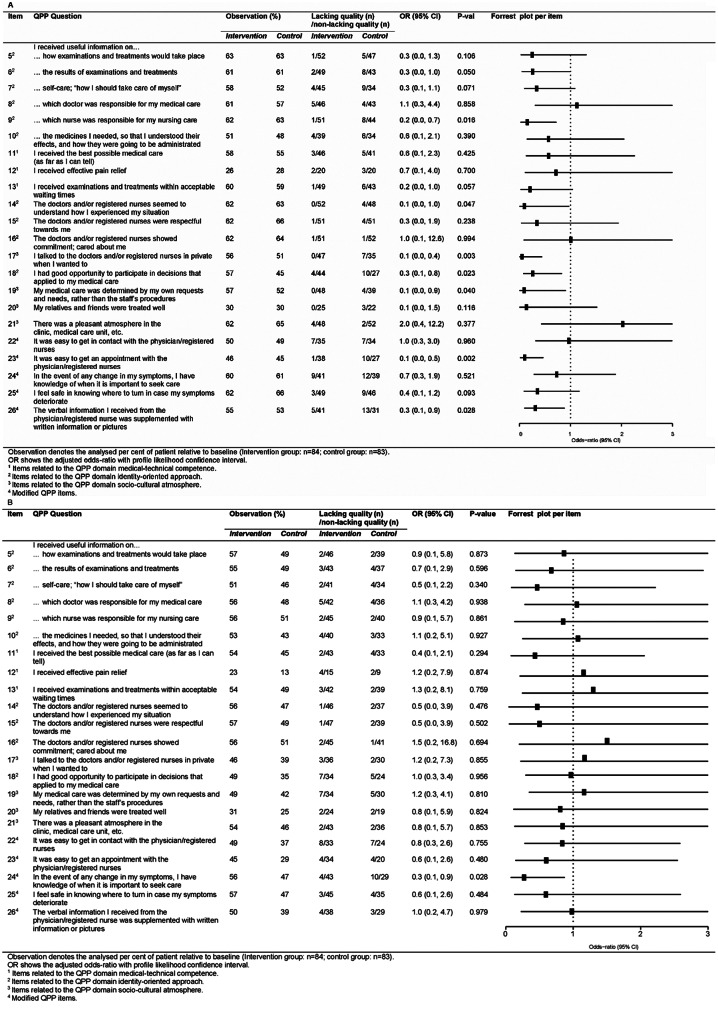
Comparison of patient-perceived ‘lacking quality’ between intervention study groups per QPP item. (**A**) 12 months and (**B**) 24 months follow-up. Odds-ratio of ‘lacking quality’. Squares denote point estimates and error bars their 95% confidence interval. The dotted vertical line indicates odds ratio = 1. Odds ratio below one proves a positive effect; odds ratio larger than one demonstrates a negative effect of the intervention

The original article [1] has been corrected.
