# Supplementary material for: Patient perceived quality of cirrhosis care– adjunctive nurse-based care versus standard medical care: a pragmatic multicentre randomised controlled study
Source: BMC Nurs. 2024 Apr 19;23:251. doi: 10.1186/s12912-024-01934-9 (PMC11027520; doi:10.1186/s12912-024-01934-9)
Supplement: Supplementary file 1 — Supplementary Material 1 [file 12912_2024_1934_MOESM1_ESM.pdf]

Additional file 2: Questionnaire Quality of Care from the Patient's Perspective

**Instructions: Fill in both A and B for each question**

| Item | | **A. This is what I experienced** | | | | | **B. This is how important it was for me** | | | | |
|---|---|---|---|---|---|---|---|---|---|---|---|
| | | Fully agree | Mostly agree | Partly agree | Do not agree at all | Not applicable | Of very high importance | Of great importance | Of rather great importance | Of little or no importance | Not applicable |
| I received useful information on | | | | | | | | | | | |
| 5 | how examinations and treatments would take place | ☐ | ☐ | ☐ | ☐ | ☐ | ☐ | ☐ | ☐ | ☐ | ☐ |
| 6 | the results of examinations and treatments | ☐ | ☐ | ☐ | ☐ | ☐ | ☐ | ☐ | ☐ | ☐ | ☐ |
| 7 | self-care; "how I should take care of myself" | ☐ | ☐ | ☐ | ☐ | ☐ | ☐ | ☐ | ☐ | ☐ | ☐ |
| 8 | which doctor was responsible for my medical care | ☐ | ☐ | ☐ | ☐ | ☐ | ☐ | ☐ | ☐ | ☐ | ☐ |
| 9 | which nurse was responsible for my nursing care | ☐ | ☐ | ☐ | ☐ | ☐ | ☐ | ☐ | ☐ | ☐ | ☐ |
| 10 | the medicines I needed, so that I understood their effects, and how they were going to be administrated | ☐ | ☐ | ☐ | ☐ | ☐ | ☐ | ☐ | ☐ | ☐ | ☐ |
| I received | | | | | | | | | | | |
| 11 | the best possible medical care (as far as I can tell) | ☐ | ☐ | ☐ | ☐ | ☐ | ☐ | ☐ | ☐ | ☐ | ☐ |
| 12 | effective pain relief | ☐ | ☐ | ☐ | ☐ | ☐ | ☐ | ☐ | ☐ | ☐ | ☐ |

| | | | | | | | | | | | |
|---|---|---|---|---|---|---|---|---|---|---|---|
| 13 | examinations and treatments within acceptable waiting times | ☐ | ☐ | ☐ | ☐ | ☐ | ☐ | ☐ | ☐ | ☐ | ☐ |
| The doctors and/or registered nurses | | ☐ | ☐ | ☐ | ☐ | ☐ | ☐ | ☐ | ☐ | ☐ | ☐ |
| 14 | seemed to understand how I experienced my situation | ☐ | ☐ | ☐ | ☐ | ☐ | ☐ | ☐ | ☐ | ☐ | ☐ |
| 15 | were respectful towards me | ☐ | ☐ | ☐ | ☐ | ☐ | ☐ | ☐ | ☐ | ☐ | ☐ |
| 16 | showed commitment; cared about me | ☐ | ☐ | ☐ | ☐ | ☐ | ☐ | ☐ | ☐ | ☐ | ☐ |
| I talked to | | | | | | | | | | | |
| 17 | the doctors and/or registered nurses in private when I wanted to | ☐ | ☐ | ☐ | ☐ | ☐ | ☐ | ☐ | ☐ | ☐ | ☐ |
| I had good opportunity | | | | | | | | | | | |
| 18 | to participate in decisions that applied to my medical care | ☐ | ☐ | ☐ | ☐ | ☐ | ☐ | ☐ | ☐ | ☐ | ☐ |
| My medical care was determined by | | | | | | | | | | | |
| 19 | my own requests and needs, rather than the staff’s procedures | ☐ | ☐ | ☐ | ☐ | ☐ | ☐ | ☐ | ☐ | ☐ | ☐ |
| My relatives and friends | | | | | | | | | | | |
| 20 | were treated well | ☐ | ☐ | ☐ | ☐ | ☐ | ☐ | ☐ | ☐ | ☐ | ☐ |
| There was | | | | | | | | | | | |
| 21 | a pleasant atmosphere in the clinic, medical care unit, etc. | ☐ | ☐ | ☐ | ☐ | ☐ | ☐ | ☐ | ☐ | ☐ | ☐ |
| It was | | | | | | | | | | | |

| | | | | | | | | | | | |
|---|---|---|---|---|---|---|---|---|---|---|---|
| 22 | easy to get in contact with the physician/registered nurses | □ | □ | □ | □ | □ | □ | □ | □ | □ | □ |
| 23 | easy to get an appointment with the physician/ registered nurses | □ | □ | □ | □ | □ | □ | □ | □ | □ | □ |
| 24 | In the event of any change in my symptoms, I have knowledge of when it is important to seek care | □ | □ | □ | □ | □ | □ | □ | □ | □ | □ |
| 25 | I feel safe in knowing where to turn in case my symptoms deteriorate | □ | □ | □ | □ | □ | □ | □ | □ | □ | □ |
| 26 | The verbal information I received from the physician/registered nurse was supplemented with written information or pictures | □ | □ | □ | □ | □ | □ | □ | □ | □ | □ |
