# Supplementary material for: Patient perceived quality of cirrhosis care– adjunctive nurse-based care versus standard medical care: a pragmatic multicentre randomised controlled study
Source: BMC Nurs. 2024 Apr 19;23:251. doi: 10.1186/s12912-024-01934-9 (PMC11027520; doi:10.1186/s12912-024-01934-9)
Supplement: Supplementary file 2 — Supplementary Material 2 [file 12912_2024_1934_MOESM2_ESM.pdf]

Additional file 3: Firth's logistic regression of QPP for 12 and 24 months, respectively

Supplementary Table 3A: QPP 12 months (items 5-15)

	QPP 5	QPP 6	QPP 7	QPP 8	QPP 9	QPP 10	QPP 11	QPP 12	QPP 13	QPP 14	QPP 15
(Intercept)	−2.16***	−1.90***	−0.78	−2.09***	−1.80***	−0.99+	−1.91***	−1.39+	−2.07***	−1.96***	−2.85***
	(0.68)	(0.58)	(0.49)	(0.62)	(0.58)	(0.57)	(0.63)	(0.77)	(0.67)	(0.66)	(0.84)
Intervention	−1.38	−1.35+	−1.08+	0.12	−1.86*	−0.56	−0.56	−0.34	−1.56+	−2.30*	−1.07
	(0.91)	(0.73)	(0.62)	(0.66)	(0.89)	(0.65)	(0.70)	(0.86)	(0.90)	(1.43)	(0.93)
Compensated	0.10	0.48	−0.82	−0.22	0.29	−0.99	−0.11	−0.48	0.33	−0.58	0.71
	(0.80)	(0.68)	(0.59)	(0.66)	(0.70)	(0.65)	(0.70)	(0.87)	(0.78)	(0.89)	(0.93)
Num.Obs.	105	102	92	98	104	83	95	45	99	104	107
Log.Lik.	-20.35	-28.59	-32.81	-28.16	-25.76	-27.28	-25.46	-14.57	-21.84	-14.05	-18.06

Estimates based on Firth's penalised logistic regression. + $p < 0.1$, * $p < 0.05$, ** $p < 0.01$, *** $p < 0.001$.

Supplementary Table 3B: QPP 12 months (items 16-26)

	QPP 16	QPP 17	QPP 18	QPP 19	QPP 20	QPP 21	QPP 22	QPP 23	QPP 24	QPP 25	QPP 26
(Intercept)	−3.20***	−1.83**	−0.75	−1.90***	−1.35*	−3.70***	−1.47**	−0.71	−1.47***	−1.75***	−0.57
	(0.95)	(0.67)	(0.51)	(0.66)	(0.69)	(0.97)	(0.51)	(0.52)	(0.48)	(0.53)	(0.45)
Intervention	0.009	−2.96**	−1.34*	−2.37*	−1.98	0.71	−0.03	−2.28**	−0.31	−1.05+	−1.17*
	(1.09)	(1.43)	(0.61)	(1.43)	(1.45)	(0.79)	(0.56)	(0.89)	(0.48)	(0.65)	(0.55)
Compensated	−0.36	0.51	−0.32	−0.36	−0.86	1.01	−0.05	−0.38	0.51	0.30	−0.44
	(1.09)	(0.80)	(0.59)	(0.90)	(1.04)	(0.92)	(0.57)	(0.66)	(0.51)	(0.62)	(0.53)
Num.Obs.	105	89	85	91	50	106	83	76	101	107	90
Log.Lik.	-9.82	-18.46	-33.06	-13.37	-9.29	-20.71	-35.34	-24.63	-48.08	-33.8	-39.73

Estimates based on Firth's penalised logistic regression. + $p < 0.1$, * $p < 0.05$, ** $p < 0.01$, *** $p < 0.001$.

Supplementary Table 3C: QPP 24 months (items 5-15)

	QPP 5	QPP 6	QPP 7	QPP 8	QPP 9	QPP 10	QPP 11	QPP 12	QPP 13	QPP 14	QPP 15
(Intercept)	−2.49***	−2.03***	−1.79**	−2.58***	−3.02***	−1.97***	−1.95**	−0.80	−2.65***	−1.71**	−2.74***
	(0.76)	(0.63)	(0.63)	(0.74)	(0.89)	(0.68)	(0.71)	(0.78)	(0.77)	(0.68)	(0.87)
Intervention	−0.15	−0.39	−0.76	0.05	−0.16	0.07	−0.84	0.14	0.26	−0.76	−0.69
	(0.88)	(0.73)	(0.80)	(0.66)	(0.88)	(0.73)	(0.80)	(0.89)	(0.83)	(1.02)	(1.00)
Compensated	−0.34	−0.08	−0.34	0.78	0.55	−0.39	−0.0008	−1.05	−0.05	−2.49*	0.16
	(0.88)	(0.73)	(0.77)	(0.75)	(0.96)	(0.73)	(0.80)	(0.86)	(0.83)	(1.44)	(1.00)
Num.Obs.	89	87	81	87	89	80	82	30	86	86	89
Log.Lik.	-20.35	-28.59	-32.81	-28.16	-25.76	-27.28	-25.46	-14.57	-21.84	-14.05	-18.06

Estimates based on Firth's penalised logistic regression. + $p < 0.1$, * $p < 0.05$, ** $p < 0.01$, *** $p < 0.001$.

Supplementary Table 3D: QPP 24 months (items 16-26)

	QPP 16	QPP 17	QPP 18	QPP 19	QPP 20	QPP 21	QPP 22	QPP 23	QPP 24	QPP 25	QPP 26
(Intercept)	−3.31***	−2.37***	−1.40*	−2.51***	−3.28***	−2.89***	−0.98*	−1.46*	−1.71***	−2.32***	−2.00***
	(0.99)	(0.79)	(0.59)	(0.73)	(1.44)	(0.91)	(0.51)	(0.70)	(0.59)	(0.71)	(0.70)
Intervention	0.41	0.16	−0.03	0.15	−0.21	−0.17	−0.18	−0.51	−1.31*	−0.52	−0.02
	(1.00)	(0.83)	(0.61)	(0.62)	(0.92)	(0.88)	(0.56)	(0.71)	(0.62)	(0.73)	(0.73)
Compensated	0.17	−0.09	−0.10	1.23+	1.66	0.46	−0.32	−0.03	1.12+	0.51	−0.13
	(1.00)	(0.83)	(0.61)	(0.73)	(1.45)	(0.96)	(0.56)	(0.73)	(0.66)	(0.78)	(0.74)
Num.Obs.	89	71	70	76	47	83	72	62	86	87	74
Log.Lik.	-20.35	-28.59	-32.81	-28.16	-25.76	-27.28	-25.46	-14.57	-21.84	-14.05	-18.06

Estimates based on Firth's penalised logistic regression. + $p < 0.1$, * $p < 0.05$, ** $p < 0.01$, *** $p < 0.001$.
